# A Report of At-Scale Distribution of Chlorhexidine Digluconate 7.1% Gel for Newborn Cord Care to 36,404 Newborns in Sokoto State, Nigeria: Initial Lessons Learned

**DOI:** 10.1371/journal.pone.0134040

**Published:** 2015-07-30

**Authors:** Nosakhare Orobaton, Dele Abegunde, Kamil Shoretire, Jumare Abdulazeez, Bolaji Fapohunda, Goli Lamiri, Abubakar Maishanu, Akeem Ganiyu, Eric Ndifon, Ringpon Gwamzhi, Matthew Osborne-Smith

**Affiliations:** 1 United States Agency for International Development/Targeted States High Impact Project, Sokoto Nigeria; 2 Jhpiego, Baltimore, MD, United States of America; 3 John Snow, Inc. Research & Training Institute, Boston, MA, United States of America; 4 European Union Support to Immunization Governance in Nigeria (EU-SIGN Project), Abuja, Nigeria; 5 Partners in Health, Boston, MA, United States of America; NIH, UNITED STATES

## Abstract

**Background:**

With an annual estimated 276,000 neonatal deaths, Nigeria has the second highest of any country in the world. Global progress in accelerating neonatal deaths is hinged to scaled-up interventions in Nigeria. We used routine data of chlorhexidine digluconate 7.1% gel utilized by 36,404 newborns delivered by 36,370 mothers, to study lessons associated with at-scale distribution in Sokoto State, North West Nigeria.

**Methods and Findings:**

Under state government leadership, a community-based distribution system overseen by 244 ward development committees and over 3,440 community-based health volunteers and community drug keepers, was activated to deliver two locally stored medicines to women when labor commenced. Newborns and their mothers were tracked through 28 days and 42 days respectively, including verbal autopsy results. 36,404 or 26.3% of expected newborns received the gel from April 2013 to December 2013 throughout all 244 wards in the State. 99.97% of newborns survived past 28 days. There were 124 pre-verified neonatal deaths reported. Upon verification using verbal autopsy procedures, 76 deaths were stillborn and 48 were previously live births. Among the previous 48 live births, the main causes of death were sepsis (40%), asphyxia (29%) and prematurity (8%). Underuse of logistics management information by government in procurement decisions and not accounting for differences in LGA population sizes during commodity distribution, severely limited program scalability.

**Conclusions:**

Enhancements in the predictable availability and supply of chlorhexidine digluconate 7.1% gel to communities through better, evidence-based logistics management by the state public sector will most likely dramatically increase program scalability. Infections as a cause of mortality in babies delivered in home settings may be much higher than previously conceived. In tandem with high prevalence of stillborn deaths, delivery, interventions designed to increase mothers’ timely and regular use of quality antenatal care, and increased facility-based based delivery, need urgent attention. We call for accelerated investments in community health volunteer programs and the requisite community measurement systems to better track coverage. We also advocate for the development, refinement and use of routine community-based verbal autopsies to track newborn and maternal survival.

## Introduction

Despite the decade-long push of the Millennium Development Goals, global progress to dramatically reduce neonatal mortality rates has been less than satisfactory. Whereas neonatal mortality rates declined globally between 1990 and 2012 by one third to 21 deaths for every thousand live births, the pace of decline is now slower than post-neonatal mortality rates during the same period [1]. More than half of the estimated 2.9 million neonatal deaths in the world in 2013 were concentrated in India, Nigeria, Pakistan, China and Democratic Republic of Congo [2]. Nigeria’s estimated annual burden of 276,000 neonatal deaths is the second highest of any country in the world [2]. Consequently, further global progress in neonatal mortality decline is tied to quicker progress in Nigeria. In 2014, the chief causes of neonatal deaths in Nigeria were severe infections (36%), complications of asphyxia (28%), preterm (27%) and others, including congenital abnormalities [2] [3]. As a stratagem to further drive down neonatal mortality rates, the deployment of chlorhexidine digluconate 7.1% an antiseptic, as a scalable intervention to prevent infections of the newborn umbilical cord, has emerged as one of the most promising innovations for newborn health in 2013[4].

A meta-analysis of three community-based randomized control trials found that chlorhexidine digluconate 7.1% gel when hygienically applied to the newborn umbilical cord in community settings resulted in a statistically significant 23% reduction in the risk of neonatal deaths [2, 3]. The World Health Organization (WHO) recently recommended its use particularly for births in home settings (WHO recommendations on postnatal care of the mother and newborn) [5–8]. The Nigeria Federal Ministry of Health has adopted the WHO guidelines[9].

A cluster-randomized efficacy trial of chlorhexidine digluconate 7.1% for newborn cord care administered by community-based health workers in rural north-eastern Bangladesh with a population of 545,000 and 13,000 births per annum, reported an 87% coverage rate among 29,760 neonates enrolled into the study[10]. These results were not surprising in such a controlled, cost-intensive setting that would be difficult to reproduce at scale in low resource settings. We did not find any published accounts of experiences and lessons about the scale up of chlorhexidine digluconate 7.1%for newborn cord care. Global experts now strongly recommend fully scaled-up programs to maximize gains on newborn lives saved [11].

This paper is a report of the implementation of the at-scale, statewide community-based distribution of chlorhexidine digluconate 7.1% gel for newborn cord care to newborns in all 23 Local Government Areas (LGA) in Sokoto State, Northern Nigeria. The findings were derived from data analyses of outcomes of 36,404 newborns that had had or had on hand a one-time application of chlorhexidine digluconate 7.1% gel applied to the umbilical cord at or around the time of birth between April and December 2013. It is hoped that these findings, to the best of our knowledge, the first of its kind to be published, will enhance our understanding of issues related to program scale up in health systems-constrained settings. It is also hoped that the paper will contribute to policy strategy dialogue on community-based approaches to assist Nigeria to ramp up implementation to narrow the gap towards the Millennium Development Goal 4 by 2015.

## Materials and Methods

### The Program Setting

Sokoto State is located in the North West Zone of Nigeria and has an area of 25,973 square kilometers. It is bordered in the north by the Republic of Niger and by the Republic of Benin in the west. Its southern and southeastern borders are shared with the Nigerian states of Zamfara and Birnin Kebbi respectively [12]. Although no published neonatal mortality rates for Sokoto State were available, the 2013 Nigeria Health and Demographic Survey (NHDS) reported a figure of 44 per 1,000 live births for the North West Zone; the reported national neonatal rate was 37 per 1,000 live births [13]. In Sokoto State, 14% of mothers reported that they received tetanus toxoid during their antenatal care visits. Less than 18% of mothers had ever attended antenatal care, 94.1% of mothers reported that they birthed their babies at home and just over 5% of births were attended in facility settings by skilled health personnel [13]. Regarding the care of the umbilical cord among non-institutional live births in Nigeria, according to the 2013 NDHS, the practice of applying something on the cord was nearly universal. Methylated spirit was applied in 30% of live births, another 31% used oil, and another 28% applied either toothpaste, ash or unspecified ointments/powder [13]. In 2% of live births, animal dung was applied to the umbilical cord [13].

Sokoto State is made up of 244 political wards. A ward is the smallest unit of self-governance in Nigeria and it is administered by unpaid community-appointed volunteers that constitute a seven-person ward development committee (WDC) led by a chairman. A ward head was often a traditional ruler, appointed by the state government [14]. The WDC typically includes the ward head that serves as the chairman and the Chief Imam of the local mosque. It unites the traditional system with the religious/spiritual and is focused on the development aspirations of the community. The WDC was recognized in the recently passed Nigeria Health Law as a foundational component of the national health system [15]. The WDC system was designed as a leadership and mobilization platform to plan, govern and sustain local ownership and participation in community development initiatives [16] [17]. Insofar as health was concerned, the main functions of WDC include active involvement in the monitoring of its revolving drug fund, supervision of community-based health volunteers (CBHVs) and oversight of health facilities including monitoring the inventory of equipment [17]. A ward is a unit of village areas, which make up districts, which in turn constitute a local government area system, the third tier of country’s administrative system operated by elected officials.

Since 2010, the United States Agency for International Development (USAID)-Targeted States High Impact Project (TSHIP) provided technical resources to strengthen the capabilities of WDC members in areas such as organizational development, rural participatory methodologies, leadership and recordkeeping, which have enhanced communities’ capabilities to more competently oversee the management of health and development services in their jurisdictions. In effect, strengthened WDC were the fulcra around which the nascent community-based health services program were developed in Sokoto State, to deliver on the community-based distribution of chlorhexidine digluconate 7.1% gel.

In a given ward, the community-based distribution system used to deliver chlorhexidine digluconate 7.1% gel for newborn cord care and misoprostol tablets for the prevention of postpartum hemorrhage to women and newborns at the household level, is comprised of a community-based health volunteer (CBHV), a community drug keeper (CDK) and a health worker based in the catchment health facility for the ward. The CBHV concept was derived from operational guidelines of the Nigeria National Primary Health Care Development Agency (NPHCDA). CBHV reside in the communities where they operate and were responsible for counseling mothers on a set of essential health messages in maternal, newborn and child health (MNCH) such as promotion of vaccination, promotion of early and regular clinic attendance for antenatal care and the promotion of skilled delivery assistance. In 2010, USAID-TSHIP and officials of the 23 LGA councils worked with the WDC and derived criteria used to select 10 CBHV per ward totaling 2,440 CBHV. The selected CBHV had to be female, reside in the ward-area, had a history of being reliable and were respected. It was an added advantage if the candidate had prior direct experience in the management of home deliveries. In the end, a typical, selected volunteer was female, Muslim, married or widowed and in the 25–55 years age bracket. Two out of three had had prior or active experience as traditional birth attendants.

CBHV were trained under the auspices of Sokoto State Ministry of Health for 10 days on 20 key health behaviors, counseling techniques and recordkeeping. They were trained for one additional day on chlorhexidine digluconate 7.1% gel administration in October 2012 after a firmed decision by Sokoto State Government to implement community-based distribution of chlorhexidine digluconate 7.1% gel with misoprostol tablets to prevent postpartum hemorrhage. The training included the hygienic, topical application of chlorhexidine digluconate 7.1% gel to the newborn cord. Counseling cards inspired by the USAID/JSI Research & Training Institute, Inc. (JSI)-supported Government of Nepal’s chlorhexidine digluconate 7.1% gel distribution experience were adapted to the Nigeria context, pretested, translated into Hausa and were deployed for use by CBHV after training [18, 19]. CBHV used pictogram forms to track and report on the number and types of counseling encounters they had in a given month. Monthly review meetings were held regularly between CBHV and supervising ward-based health workers during which time refresher training was provided alongside peer-to-peer learning. The guidelines of the NPHCDA recommended that CBHV be paid monthly stipend although no amount was specified. In the Sokoto State program, a monthly stipend of NGN2000 (equivalent to 2014 US$ 16) was paid to each CBHV to cover transport costs, and borne by USAID/TSHIP at the time of this writing. The low stipend amount in this program was considered more financially sustainable and therefore more likely to be adopted by State and LGA decision makers.

At the same time as the state government decided to procure these medicines for community-based distribution, it embarked on a systematic and comprehensive campaign that prepared communities and citizens to demand them. The lot of organizing an advocacy campaign fell on the MOLG. From the onset, it pronounced that community distribution would commence only after the MOLG had received sufficient feedback through its network of district heads that communities positively welcomed the program, and had no opposition to it. On its own accord, MOLG developed and mass produced briefings recorded on compact discs that explained the benefits chlorhexidine digluconate 7.1% gel and misoprostol in *Hausa*, the local language, which were distributed to all TV, radio—public, private and community—stations; these were regularly aired for at least four months. Live, call-in TV discussion programs that included Islamic scholars and government officials were regularly held over a five-month period. The MOLG used its monthly meetings with chairmen of LGA to provide multiple, monthly briefings on the program and its objectives. In turn, each LGA chairman briefed heads of departments and district heads under their jurisdictions. At the same time, Sokoto State Chairman of Jama’atu Nasril Islam/ Nigeria AID Group, and Nigeria’s largest Islamic nonprofit organization, spearheaded an advocacy campaign with local government officials, clerics and Imams. Clerics and Imams in turn used the pulpit during Friday *Juma’at* services and explained the program to men, whose consent was co-essential to the program’s success. In addition, imams spread the word during naming ceremonies and wedding *fatihas*. WDC chairmen in the 244 wards were consulted and briefed extensively. A key consideration in the advocacy was to detect discern for potential sources of opposition or concerns, and to make certain that they in turn were respectfully and thoroughly addressed. All in all, these combinations of advocacy actions spanned a period of five months. We also stress that several of these advocacy and consultative events were focused preponderantly on men, whose unequivocal support both to the concept and the delivery design of the program, was indispensable for the whole program to proceed unencumbered.

The CDK is a highly regarded resident appointed by the WDC and entrusted to distribute chlorhexidine digluconate 7.1% gel and a three-tablet pack of misoprostol tablets. The 1,220 CDK in the state averaged 5–7 per ward, lived within 400 meters of households and 97% were male. Community leaders insisted on this function as assurance that these medicines were used solely for the purposes intended. A two-bin container system, each bin with 15 doses of chlorhexidine digluconate 7.1% gel and 15 doses of misoprostol tablets was designed for each CDK to manage commodity supply and prevent stock-out. When supplies in the first bin are used up, the CDK was required to return to the supervising health center and restock. Each CDK received a one-day long training on the function and importance of chlorhexidine digluconate 7.1% gel, its proper storage, proper stock management, recordkeeping on stocks, on the conditions of newborn from birth to 28 days and the condition of mothers from birth to six weeks postpartum. The messages were regularly reinforced during the monthly review meetings of CDK managed by health workers in the ward area.

**Fig 1:**
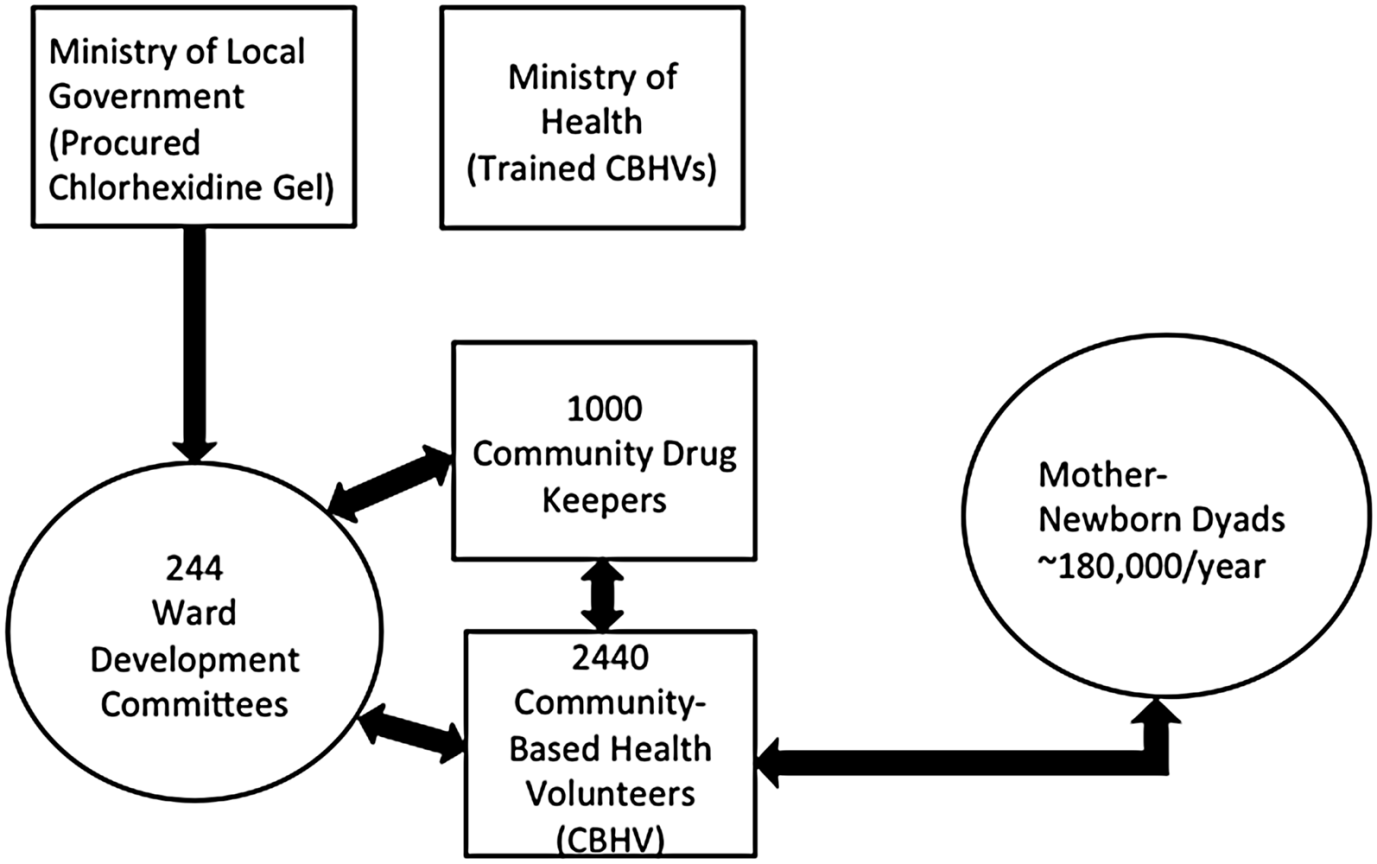
Schematic Diagram of Actors in the Delivery of Chlorhexidine Digluconate 7.1% Gel At-Scale in 244 Wards in Sokoto State, Nigeria.

Sokoto State Government through its Ministry of Local Government (MOLG) financed the purchase of chlorhexidine digluconate 7.1% gel utilized in this scale up exercise. This was the first instance of a government-led and financed procurement for chlorhexidine digluconate 7.1% gel in Nigeria and in Sub-Saharan Africa. An initial 56,000 units were purchased and were divided in equal lots between the 23 LGA, without regard to the population sizes of the LGA. This decision was considered a visibly demonstrative way to administratively signal that all LGA had received equal consideration under this program. As an aide to the MOLG, USAID/DELIVER and USAID/TSHIP had a priori, shared detailed quantifications with pipeline analyses based on available demographic information. The MOLG decided to distribute the bins of medicines directly to the chairmen of the 244 WDC. A WDC chairman also received a reserve supply for safekeeping at the catchment, ward health center. The MOLG delivered the supplies directly to WDC chairmen, and in effect transferred the ownership and control of this program to the community. WDC in turn handed over the pre-packaged supplies directly to the CDK in their respective wards. A schematic diagram of the relationships between WDC, CBHV, CDK and mother-newborn dyads is shown in Fig 1.

### The Chlorhexidine Digluconate 7.1% Gel Distribution Program

Chlorhexidine digluconate 7.1% gel 3 gram tubes along with misoprostol tablets were dispensed by CDK primarily to a CBHV at the same time on demand, triggered by notification that a woman in his jurisdiction was in active labor. The CBHV who closely monitored pregnant women in her area was the principal notifying person. CBHV were expected to have counseled expectant mothers on the proper administration of chlorhexidine digluconate 7.1% gel and the mandatory care of the cord in the first 24 hours and beyond. CBHV delivered medicines obtained from CDK to mothers, and directly observed mothers as they ingested misoprostol tablets. CBHV were often requested to directly apply chlorhexidine gel on the freshly cut newborn cord.

As soon as chlorhexidine digluconate 7.1% gel and misoprostol tablets were dispensed to a mother in labor, her name, home address and the date it was dispensed was entered into an “outcome” form maintained by the CDK. The CDK worked closely with the CBHV who followed-up on mother and newborn and verbally reported back information on whether the newborn was born alive or not. This information was also entered into the “outcome” form. All the deaths within the first 28 days of life—around birth, at or before 7 days and thereafter–-were also recorded. The CBHV also reported back on mothers who were referred to the nearest health facility for excessive bleeding and other complications; this too was recorded in the “outcome” form. All maternal deaths within 42 days postpartum were also recorded. In instances of multiple births to a woman, each newborn record was separately recorded and related with a given mother’s information; one unique record was produced per child and each member of a multiple birth was linked to a mother’s record. The mother’s record was entered once regardless of the number of her births.

Monthly data review meetings were held between health facility workers, CDK and CBHV. Inconsistencies in data collected were identified and addressed. Thereafter, the collated data were shared with USAID-TSHIP, which undertook additional data quality checks described below. The data was entered for each record using EpiData. The data was subsequently converted into a format that permitted analysis using STATA statistical software package.

Verbal autopsies were undertaken to establish the causes of deaths reported in the course of the routine data keeping by CDK and CBHV. This verification exercise was carried out in collaboration with National Population Commission Office in Sokoto in October 2014. The verbal autopsy process utilized a paper-based questionnaire derived from WHO and have been used extensively in the Nahuche Health and Demographic Surveillance System (HDSS) site in Zamfara State, also in the North West Zone [20]. A list of newborns that had died including their names, residential address, location by LGA and the name of the supervising CDK was extracted from the database mentioned earlier. The newborns were disaggregated into each of the three zones in the state and each zone was assigned to a team of three, one male, and one female researcher as well as an officer from the Sokoto State National Population Commission.

The verbal autopsy exercise commenced with an initial, critical courtesy visit to the Office of the Chairman of the Local Government where the dead newborn resided. Similar courtesy visits were paid to the district head and the chairman of the WDC where the child resided. Thereafter, the supervising CDK was contacted, who confirmed the exact location of the newborn’s residence, and helped to find a credible informant, to obtain pertinent information for cause of death verification. Prior to each interview, an informed consent statement was read in Hausa to the informant and the interview only commenced in earnest after informed consent was granted. If a woman informant wished to be interviewed privately in her home, a female researcher in the team did so. The interview established the informant’s relationship with the deceased, the circumstances of the mother and her delivery, care of the cord at the time of delivery and a detailed history of illness, accidents and neonatal conditions. Following the data collection, the filled-out questionnaires were given to three doctors trained in cause of death certification who worked independently and consensually assigned a cause of death to each of the deceased.

Given the non-routine nature of verbal autopsies, ethical clearance for its conduct was sought and obtained a priori from the Sokoto State Health Research Ethics Committee. All respondents had had the purpose of the study and the voluntary nature of their participation dully explained in Hausa. Written, informed consent was obtained from all respondents after explanations that they were free to choose not to participate in the study without any consequences.

### Data Quality Considerations

Several steps were taken to assure the data quality from this scale-up intervention. The first was the design of the earlier mentioned outcome form, which was intentionally structured to collect data on very few variables that were readily verifiable and manageable by CDK that possessed limited reading and writing skills. Second, the data entered was analyzed for completeness and internal consistency. Third, the records of newborns classified as dead were carefully examined. USAID-TSHIP staff and staff of Sokoto State National Population Commission visited CDK and CBHV in wards where deaths were reported and re-confirmed the deaths. Finally, a 5% random sample of 1,900 mother-newborn of the 36,404 individual newborns records in our database was revisited in their homes in 23 LGAs and spread in a total of 239 wards. Overall, a total error rate of 1.6% was found and rectified.

All data from the outcome forms and verbal autopsy interviews were entered twice, separately, and then reconciled with EpiData software. STATA was used for the data analysis.

## Results

A total of 36,404 newborns were delivered by 36,370 mothers in the data tracked from April 2013 to December 2013 throughout the 23 LGA and all 244 wards in the state. About 99% of newborns were singletons. There were 32 sets of twins and one set of triplets.

**Fig 2:**
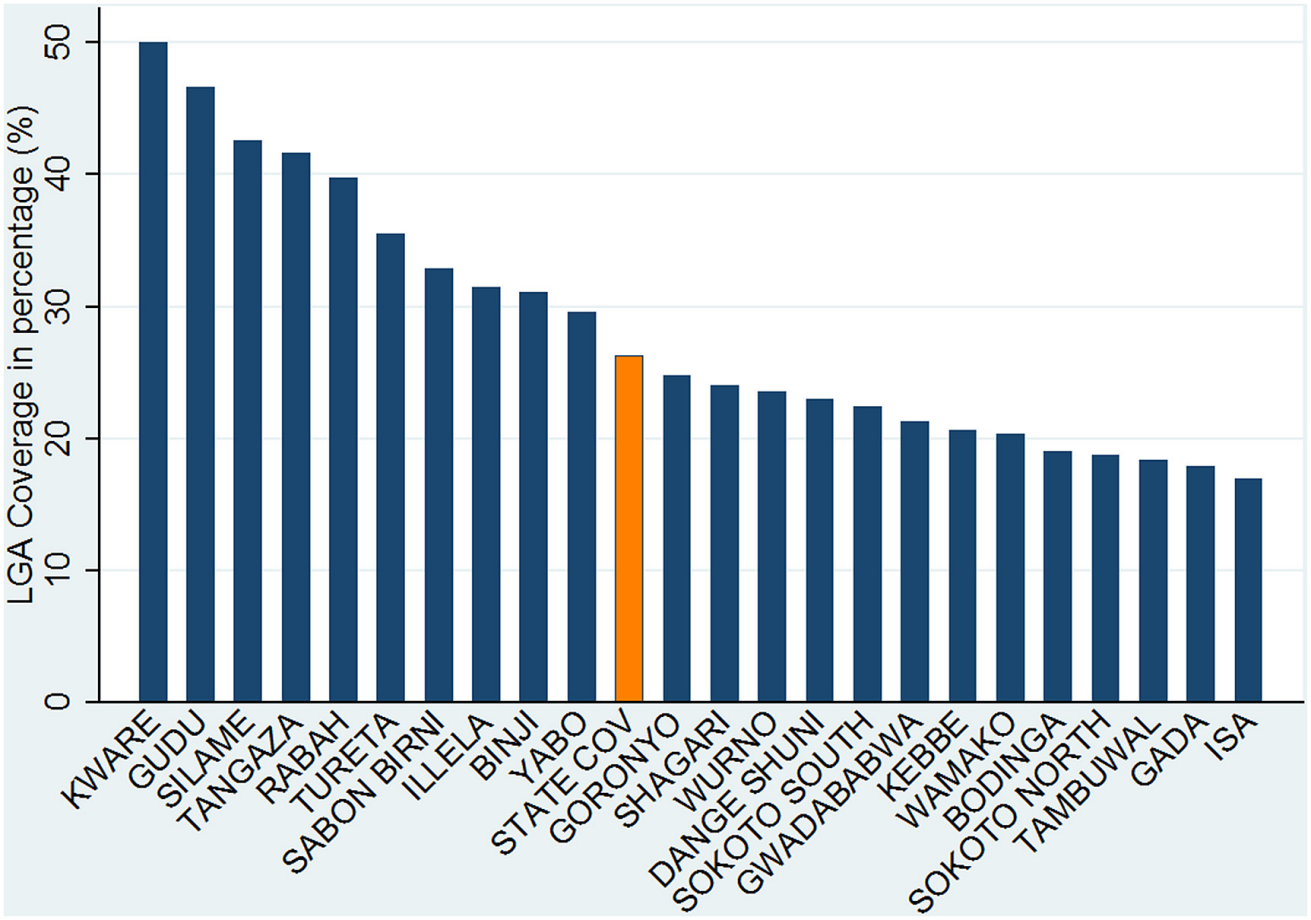
Coverage of Newborns as a Percent of Expected Births that Received Chlorhexidine Digluconate 7.1% Gel in 23 Local Governments and at State Level, April to December 2013, Sokoto State, Nigeria (N = 36,404).

To estimate the coverage of newborns that received chlorhexidine digluconate 7.1% gel, we used the number of expected births disaggregated by LGA supplied by the Sokoto State Office of the National Population Commission. There were a total of 184,255 births expected for the calendar year of 2013 [21]. The prorated expected births for a period of nine months between April and December 2013 was estimated at 138,191. As shown on Fig 2, 26.3% of all expected newborns were reached with chlorhexidine digluconate 7.1% gel. The coverage rate ranged from a low 17% in Isa LGA to 50% in Kware LGA. Five LGAs attained a coverage rate of 40% or higher. Thirteen LGA had coverage rates that fell below the 26.3% state average and the remaining 10 were above. The correlation coefficient between population of expected births in LGAs and their coverage rates was -0.71. On average, LGA with larger populations tended to have lower coverage rates and those with smaller populations had higher coverage rates. We compared the coverage rates of urban LGA with rural LGA. Rural-urban classifications of LGA were based on government-supplied information. Urban LGA tended to have lower coverage rates compared to more rural LGA. One argument was that urban LGA had greater access to secondary and tertiary hospitals and so were less likely to use the home delivery services. The coverage rate achieved in rural LGA was 28.8% and it was 20.2% for urban LGA.

**Fig 3:**
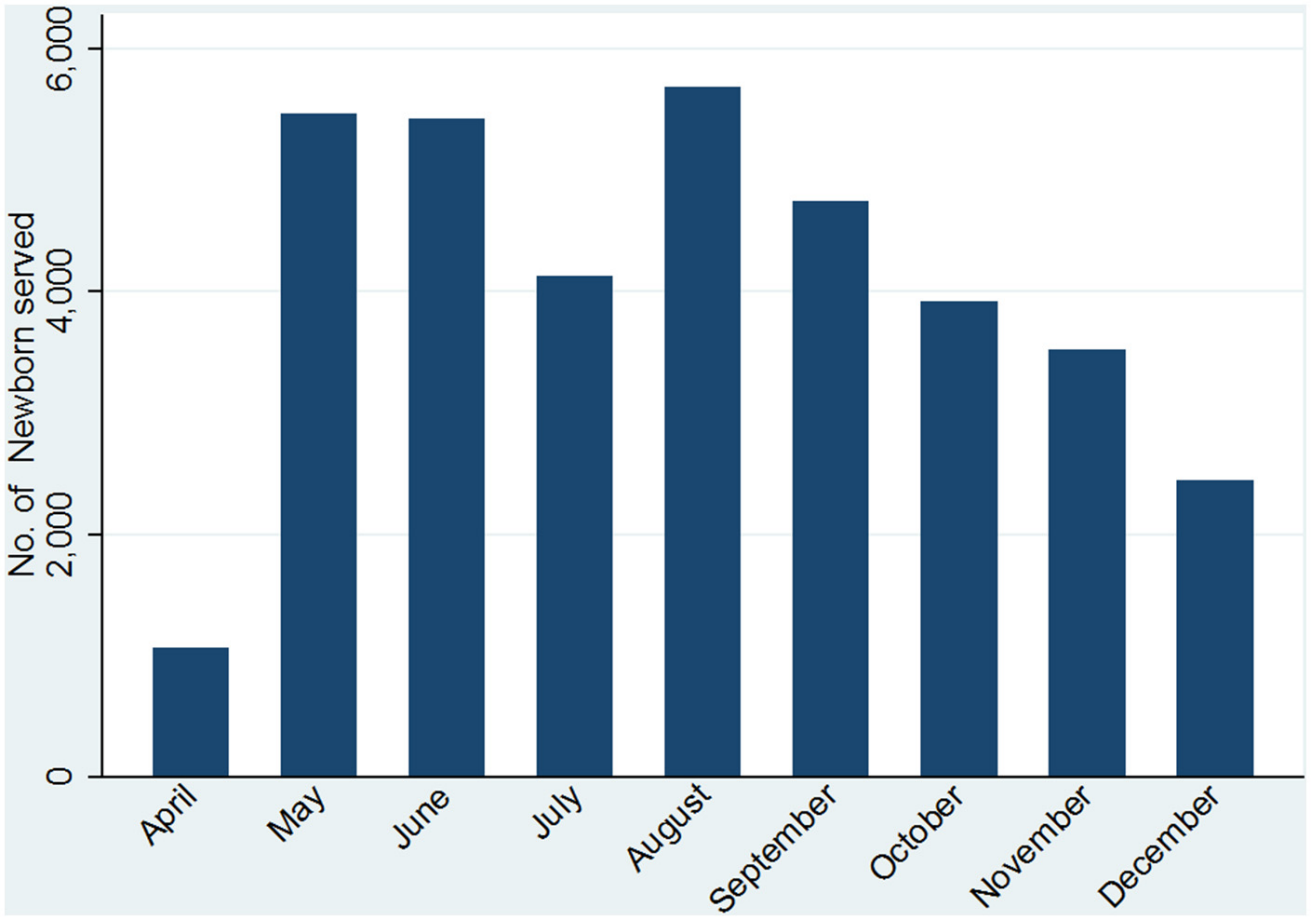
Newborns as Percent of Expected Births, Reached with Chlorhexidine 4% gel in Sokoto State, April–December 2013.

Fig 3 shows a bar chart of the monthly number of newborns that used chlorhexidine digluconate 7.1% gel throughout Sokoto State from April to December 2013. The number of newborns rose fivefold from 1,061 in April to 5,465 in May, reflecting an improved penetration of services along with awareness of services. The sharp increase also reflected the high, unmet demand for services. Assuming a constant rate of newborns for the nine-month period, 11,536 tubes of chlorhexidine digluconate 7.1% gel would be needed per month to cover all expected births. However, the highest attained level, reached in August, was 5,690 or 49% of expected monthly births. The monthly number of newborns served with chlorhexidine digluconate 7.1% gel remained at around 5,550 (48%) through to a peak of 5,690 in August, and then dropped to 2,445 (21%) in December. The tapering of quantities consumed by more than 50% from the plateau of 5,550 suggests shortages fueled by a combination of an inadequate pipeline of supplies and a problem of mal-distribution of chlorhexidine digluconate 7.1%. The MOLG officials invoked civil service regulations to rationalize on why it limited the purchase of chlorhexidine digluconate 7.1% gel to cover the needs of one quarter at a time, which did not include an adequate filling of the pipeline. In addition, the decision by the MOLG to give equal quantities of chlorhexidine digluconate 7.1% gel to LGAs without regard to population size, more likely aggravated mal-distribution.

### Tracking Neonatal Deaths

**Fig 4:**
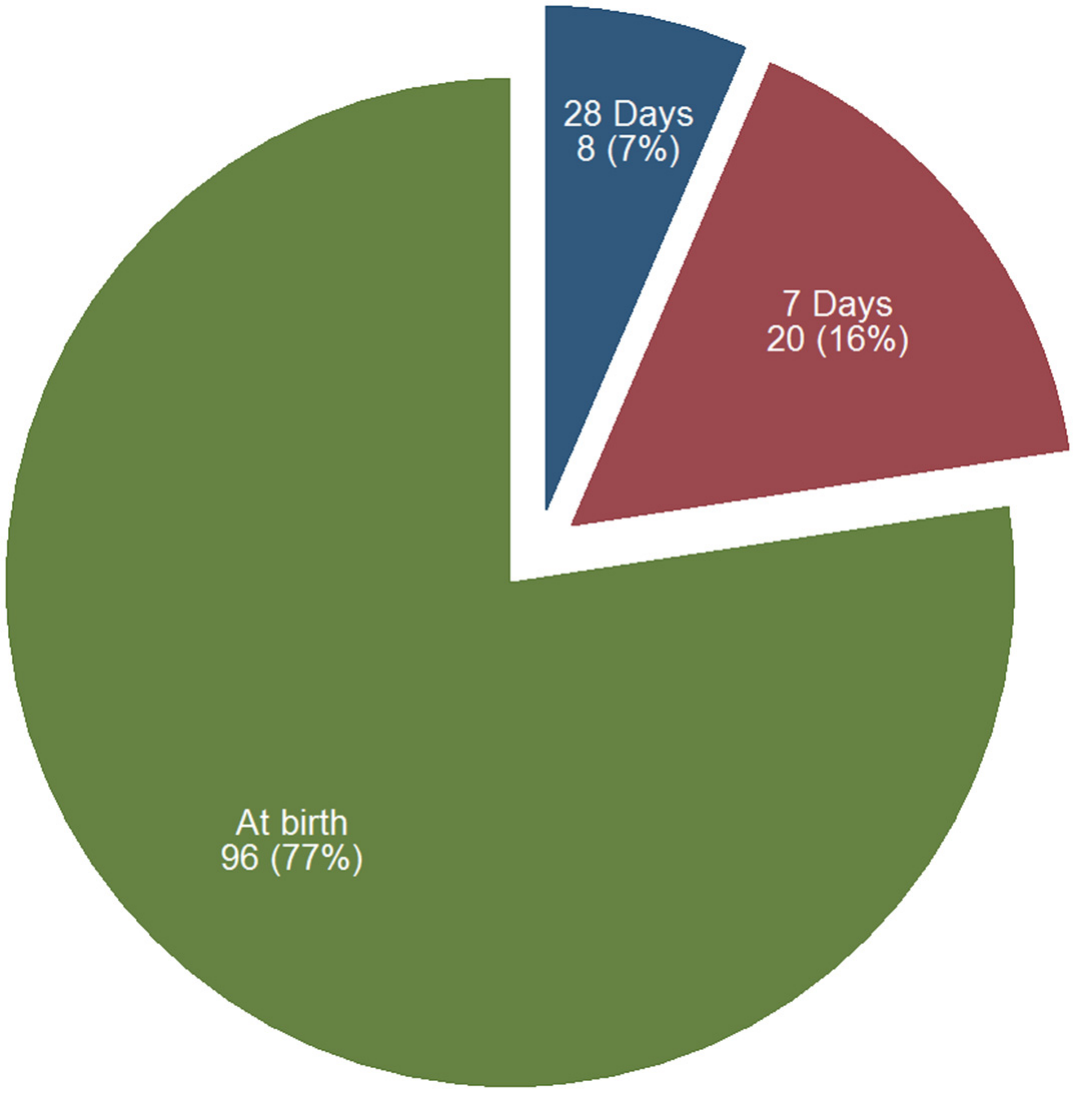
Pre-verified Neonatal Deaths (N = 124) by Reported Timing in the 36404 Cohorts that received Chlorhexidine from April–December 2013. Sokoto State, Nigeria.

Fig 4 shows the distribution of pre-verified neonatal deaths by timing of deaths for the cohort of newborns examined from April to December 2013. Of the total newborns who were born and who received a single application of chlorhexidine digluconate 7.1% gel and were followed through to 28 days, a total of 36,380 or 99.97% survived. A total of 124 confirmed deaths were recorded. At or around birth, there were 96 deaths, which represented 77% of all deaths in the neonatal period. By seven days, there were an additional 20 deaths representing 16% of the total deaths; this brought the cumulative percent of deaths to 93%. The balance of the eight deaths or 7% of deaths occurred between 8 days and 28 days postpartum. All the observed deaths were concentrated in 18 LGAs. Nearly 60% of deaths were concentrated in seven LGAs—Silame, Gada, Sabo Birni, Sokoto South, Isa, Gudu and Tureta. There were no deaths recorded in Illela, Wurno, Sokoto North, Kebbe and Yabo LGAs in the cohort. Majority of the deaths occurred in a rural LGA at 106 or 85% of all deaths. Sokoto State is divided into three senatorial zones; Sokoto East, Sokoto Central and Sokoto South. Nearly half of all deaths (61 deaths) occurred in Sokoto Central, 46 in Sokoto East and 17 in Sokoto South.

**Fig 5:**
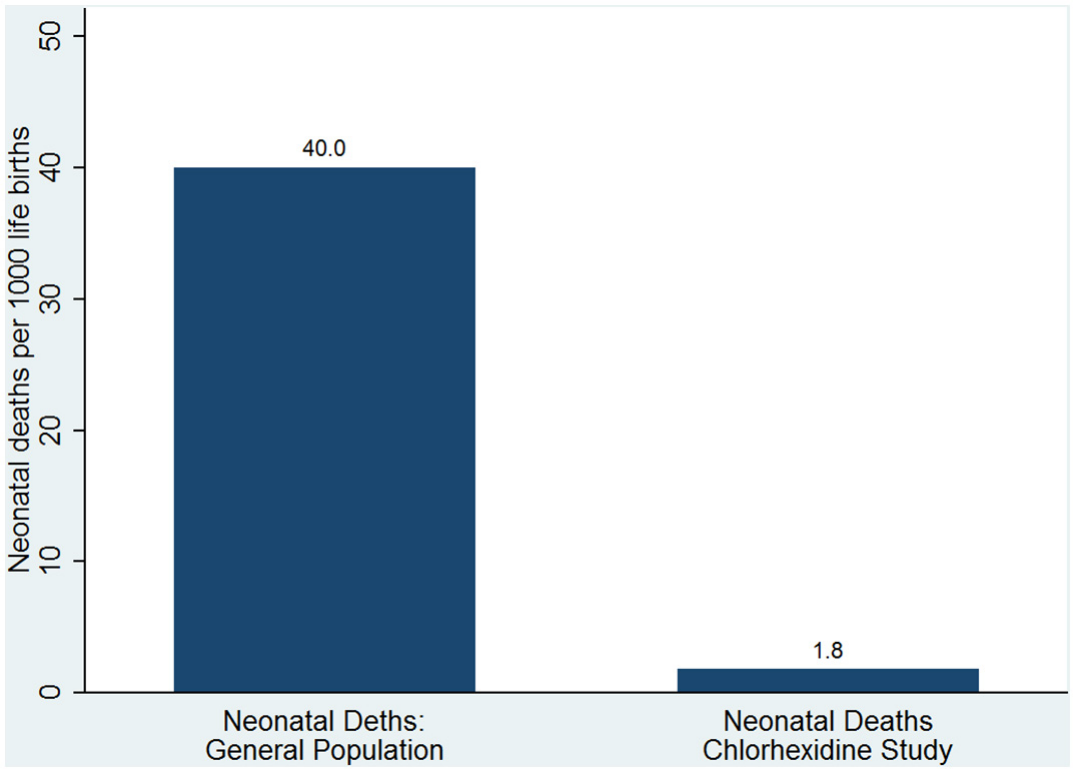
Neonatal Mortality Rates in Cohort of 36,404 Newborns April–December 2014. Sokoto State, Nigeria.

The causes of death in the 124 deaths derived from verbal autopsies are shown in Table 1. The top three causes of death that accounted for 87% of all deaths were stillbirths 76 (61%), neonatal sepsis 19 (15%) and birth asphyxia 14 (11%). The stillbirths, made up of the fresh and macerated, occurred at a ratio of 3.5 fresh stillbirths for every macerated one. The lesser occurring causes recorded included prematurity, acute respiratory infections/pneumonia, severe anemia and malnutrition and congenital causes. In excluding 76 stillbirths from the initially unverified124 neonatal deaths, the confirmed number of live births that suffered mortality in the neonatal period were a total of 48. The principal causes of death in this cohort were neonatal sepsis 19 (40%), birth asphyxia 14 (29%), other unspecified causes 5 (10%) and prematurity 4 (8%) (see Table 2). With stillbirths deducted from the denominator to obtain an adjusted number of 36,328, we obtained an annualized newborn mortality rate of 1.8 per 1,000 live births (Fig 5). This mortality rate was considerably lower than the annualized neonatal mortality rate of 44 deaths per 1,000 live births reported in the North West zone, the closest counterfactual [13], and the neonatal mortality rate of 10.4 per 1,000 live births for 2012 obtained from the Nahuche Demographic Surveillance System (HDSS) based in the Nahuche District of Bungudu LGA, Zamfara State, adjacent to Sokoto State [22].

**Table 1:** Causes of Death of 124 Deaths in Cohort of 36,404 Newborns Offered Chlorhexidine digluconate 7.1% Gel in Sokoto State, Nigeria, April-December 2014

Causes of Death	Number	Percent
Fresh Still birth	59	48%
Macerated Still birth	17	14%
Neonatal Sepsis	19	15%
Birth Asphyxia	14	11%
Other Unspecified	5	4%
Prematurity	4	3%
ARI/Pneumonia	2	2%
Malaria	1	1%
Severe Anemia	1	1%
Severe Malnutrition	1	1%
Congenital Malformation	1	1%
Total	124	100%

**Table 2:** Causes of Deaths of Live-born Neonates Among 36,404 Newborns Who Received Chlorhexidine Digluconate 7.1% Gel for Cord Care.

Neonatal Sepsis	19	40%
Birth Asphyxia	14	29%
Other Unspecified	5	10%
Prematurity	4	8%
ARI/Pneumonia	2	4%
Malaria	1	2%
Severe Anemia	1	2%
Severe Malnutrition	1	2%
Congenital Malformation	1	2%
Total	48	100%

## Discussion

We described the results of scaling up the use of chlorhexidine digluconate 7.1% gel in non-controlled, real world settings. For a medicine never previously used in Nigeria for newborn care, our experience offers some lessons. The first was that in a health system constrained with the shortage of health personnel, community health volunteers have deployable capabilities in the distribution of life-saving commodities such as chlorhexidine digluconate 7.1% gel. Second, with extensive evidence-based advocacy to and by state government officials and with the support of traditional rulers and Islamic scholars, it was possible to secure community demand for chlorhexidine digluconate 7.1% gel in Sokoto State. The third and perhaps the most critical was securing the trust of the community and its leaders to accept these medicines and to commit community structures and its’ volunteers. Fourth, the sharp uptake of chlorhexidine digluconate 7.1% gel suggested a community that was primed to use the product and such readiness is noteworthy for communities that have historically been suspicious of externally introduced medicines. The evident ownership by communities and active involvement of men as CDK most likely boosted rapid community adoption. The tactic by the government to handover the medicines directly to the WDC chairmen was a powerful lever that fostered local ownership. Islamic leaders, traditional leaders and government officials made significant upfront investments in securing community-acceptance. Fifth, the very high newborn survival rate of 99.97% suggested that a single dose application of chlorhexidine digluconate 7.1% gel and instructions on the hygienic care of the cord after application were readily implemented by women. The high survival rate also suggested, on account of the very high 95% rate of home delivery in hygiene-constrained settings, that the contribution of infections to neonatal mortality may be much higher than previously assumed. Here, chlorhexidine digluconate 7.1% gel along with hand washing replaced some harmful substances previously and typically applied to the cord. For example, in a social context where the topical application of an agent to the umbilical cord was nearly universal, the 2013 NDHS reported that 23% of recent non-institutional live births reported that toothpaste, ash or animal dung was applied to the umbilical cord across Nigeria [13]. Still, the 15% of deaths attributable to neonatal sepsis in the 48 live births underscores the importance of the need to tackle newborn sepsis as a whole in Sokoto State. It is unclear how many of these deaths were related to instances where mothers had subsequently applied other substances on the newborn cord after the first, one-time application. Our data collection instruments did not inquire about mothers’ compliance to the sole, exclusive use of the gel. The new national policy of multiple applications of chlorhexidine digluconate 7.1% gel will importantly offer additional protection to newborns. The high proportion of deaths associated with fresh and macerated stillbirths as well as birth asphyxia, reinforce the vital importance of stepping up mechanisms and incentives that will increase the use of quality antenatal care services by women and, in particular, the use of health facilities for better delivery outcomes. Notwithstanding, community distribution of chlorhexidine digluconate 7.1% gel, which is critically important in home deliveries, a call-to-action for a renewed focus for women to regularly make antenatal visits—that deliver essential medicines, that are culturally appropriate and respectful—and the use of skilled delivery assistance in facility settings, remain a compelling priority [23, 24].

There were evident problems associated with commodity supply and availability. First, the procurement decision by the government precluded a robust use of the forecasted needs despite adequate logistics planning. Second, there were delays in the arrival of the imported gel product due to delays in arrival and customs clearance, among others. There were also delays in distribution after medicines had arrived. The need for the adoption of evidence-based logistics sciences as the basis for commodity procurement planning is evident.

There were several strengths of the scale-up exercise thus far that could be leveraged further. It was a culturally relevant, people-centered, public health approach and it served a clear, perceived need of the communities. Community trust was actively cultivated and leveraged in the upfront design exercise and throughout the execution of the program. The overall design approach was strong on governance in that it involved all levels of government, especially at the ward and community levels. The approach emphasized community leadership and ownership, which portends well for sustainability. It also underscored the central importance of the regular availability of key commodities in the fight to save newborn lives. The underlying community-based distribution system helped establish a platform that consequently helped to promote a range of other essential MNCH interventions such as routine immunization, polio eradication, malaria management and the distribution of Vitamin A capsules. For example, the platform was instrumental for Sokoto State to attain Vitamin A coverage of 85% of children aged 6–59 months in June 2014 [25]. Similarly, Sokoto State Primary Health Care Development Agency and the Ministry of Health (MOH) recently instituted a system for the routine distribution of long-lasting insecticide treated nets and the community-based distribution system and network served as the backbone of the system [25]. The system also aided the initial accumulation of experience on a community-based health information system. This effort uncovered an underutilized partnership between the MOLG and the MOH. They also highlighted constraints associated with the jurisdiction of health facilities in the state and what needs to work with the component parts of a health system from the perspective of unity. All stakeholder ministry agencies recently began consultations on the adoption of a state version of a national policy of Primary Health Care Under One Roof under the leadership of the State primary health care development agency [26]. The formal inclusion of program roles for men increased men’s understanding of the powers of commodities and transformed them to more proactively advocate for and demand better MNCH with politicians.

There remain several weaknesses in the effort so far. The main inputs in terms of training resources and the provision of requisite technical assistance as well as the financing of stipends for CBHV rely heavily on donor financing. This administrative behavior of the MOLG slowed the adoption of sound policy recommendations such the use of forecasting information to guide procurement. However, it is also important to stress that in a number of instances, administrative tactics were deftly deployed and aided the faster take off of the project that in turn eliminated further delays that could have unraveled the whole program. This trade-off meant that a surviving but less than optimal program was better than no program at all. Nonetheless, commodity forecasting and procurement needs to be improved significantly. The recently installed local manufacturing capacity of chlorhexidine digluconate 7.1% gel in Nigeria will significantly reduce the delays in delivery after procurement. The capacity of LGA Monitoring and Evaluation Officers to monitor data still needs to be strengthened considerably. The shortage of skilled facility-based providers, which also spurred this intervention, still remains unresolved. Community-based interventions for MNCH ought to be additional to but not a substitute to the provision of skilled health workers. With the growing focus on community participation, additional work remains to clarify and define the roles of health centers and hospitals to better support community efforts.

### Program Limitations

The program did not have a control within Sokoto State with which to compare program impact. It was not designed to test whether or not going to scale was beneficial or not. Rather, the focus was to learn of factors and conditions to operate at scale as effectively and as efficiently as possible. In this respect, one would argue that comparing the program performance to a counterfactual would have sufficed. The program effect of administering chlorhexidine digluconate 7.1% gel in births in home settings resulted in neonatal morality rates that were considerably lower than the rate of the North West Zone and that of Nahuche District in Zamfara State, the two counterfactuals included in this analysis.

The data used in this analysis were obtained from a routine data collection system as part of a statewide community distribution program. It was collected in the context of a program setting that was not controlled and therefore reflected the challenges associated with large-scale program interventions. The CDK could have kept incomplete records. It is possible that information associated with events that occurred well after delivery might have suffered from recall bias than those that occurred at the time of the delivery. These were addressed through triangulation of information sources between the CDK, CBHV and subsequent data quality checks that followed. Particularly vulnerable to recall bias was the recording of deaths. Each of the reported deaths were revisited and verified. The risk of entering fictitious data was considered low to inconsequential because the CDK served their communities. To the best of our understanding, there were no perverse incentives at play that were likely to cause data collection to be inaccurate or incomplete. CDKs displayed high fidelity in the performance of their tasks as primary data collectors.

Although this data set was not obtained under controlled settings, we consider this a key strength in that it provided insights into what type of data collection system was realistically possible to operate with acceptable levels of precision needed to make valid statements.

## Conclusion

We have presented the early experiences and results associated with taking a community-based distribution of one of two lifesaving medicines to scale in Sokoto State. The lessons of this program have remained influential in guiding the formulation of the national approach to the nascent scale-up of chlorhexidine digluconate 7.1% gel use in Nigeria. This program effort also offered a platform that united a constellation of national policy, state financing, community acceptance and ownership with private sector investments, at the service of newborns, and it showed promise. The results revealed opportunities and challenges that have to be addressed for an eventually fully successful program impact marked by high program coverage. The role of the government remains indispensable in the scale-up of MNCH in Sokoto State. The same will most likely apply elsewhere in Nigeria. The nature of administrative behavior notably in how they affected the timeliness and completeness of procurement driven by evidence-based forecasts remained partially resolved. It is hoped that the MOLG will act and follow through with procurements that satisfied local demand over longer time horizons and also account for filling the pipelines.

The prospect of going to scale with community-based structures has opened several exciting possibilities of public health consequence, which need to be developed further and closely monitored. The first is that the community data tracking is akin to the forerunner of a whole-state surveillance system tracking and recording maternal and neonatal deaths. That a substantial increase in the survival of newborns past the neonatal period has been achieved, it has also increased the operational feasibility of the tracking, certification and registration of the causes of maternal and neonatal deaths at the community level. It is hoped that the state government will seize this opportunity to develop a stronger platform for death registration and death certification. In an effort to redress weak, untapped partnerships between the MOH, the MOLG and others, Sokoto State have taken initial steps towards the coordination of its primary health care under the aegis of a SPHCDA. The intent of the coordination must at least include the consolidation of health centers and dispensaries under one common authority. The commitment towards the policy of multiple applications of chlorhexidine digluconate 7.1% gel for newborns should hold sway and still needs to be vigorously advocated. However, some state officials in Sokoto were wary that the abandonment of the practice of single application already introduced in the state in favor of multiple applications would increase overall costs and consequently reduce the attractiveness of the program. Perhaps of greater concern, is the fragility of community trust. The recent memory of the loss of trust in polio programs and high cost in time lost and money to regain this community trust was still fresh in the minds of government officials [27]. Therefore, officials have considered it a moderate to high risk in Sokoto to switch gel tube size and introduce multiple application of chlorhexidine digluconate 7.1% gel. Policy dialogue managed by federal health authorities is urgently needed to resolve and permanently shift the move from single use in the state and elsewhere to multiple applications.
